# Natural Products Diversity in Plant-Insect Interaction between *Tithonia diversifolia* (Asteraceae) and *Chlosyne lacinia* (Nymphalidae)

**DOI:** 10.3390/molecules24173118

**Published:** 2019-08-28

**Authors:** Marília Elias Gallon, Eduardo Afonso Silva-Junior, Juliano Geraldo Amaral, Norberto Peporine Lopes, Leonardo Gobbo-Neto

**Affiliations:** 1 Núcleo de Pesquisa em Produtos Naturais e Sintéticos, School of Pharmaceutical Sciences of Ribeirão Preto, University of São Paulo, Av. do Café s/n°, Ribeirão Preto, SP 14040-903, Brazil; 2 Centro Universitário do Vale do Araguaia, R. Moreira Cabral, Barra do Garças, MT 78600-000, Brazil; 3 Instituto Multidisciplinar em Saúde–Campus Anísio Teixeira, Universidade Federal da Bahia, R. Hormindo Barros, 58, Qd 17, Lt 58, Vitória da Conquista, BA 45029-094, Brazil

**Keywords:** Heliantheae, LC-MS, metabolomics, molecular networking, chemical ecology, sesquiterpene lactones, flavonoids, lysophospholipids

## Abstract

The chemical ecology of plant-insect interactions has been driving our understanding of ecosystem evolution into a more comprehensive context. *Chlosyne lacinia* (Lepidoptera: Nymphalidae) is an olygophagous insect herbivore, which mainly uses host plants of Heliantheae tribe (Asteraceae). Herein, plant-insect interaction between *Tithonia diversifolia* (Heliantheae) and *Chlosyne lacinia* was investigated by means of untargeted LC-MS/MS based metabolomics and molecular networking, which aims to explore its inherent chemical diversity. *C. lacinia* larvae that were fed with *T. diversifolia* leaves developed until fifth instar and completed metamorphosis to the adult phase. Sesquiterpene lactones (STL), flavonoids, and lipid derivatives were putatively annotated in *T. diversifolia* (leaves and non-consumed abaxial surface) and *C. lacinia* (feces, larvae, pupae, butterflies, and eggs) samples. We found that several furanoheliangolide-type STL that were detected in *T. diversifolia* were ingested and excreted in their intact form by *C. lacinia* larvae. Hence, *C. lacinia* caterpillars may have, over the years, developed tolerance mechanisms for STL throughout effective barriers in their digestive canal. Flavonoid aglycones were mainly found in *T. diversifolia* samples, while their glycosides were mostly detected in *C. lacinia* feces, which indicated that the main mechanism for excreting the consumed flavonoids was through their glycosylation. Moreover, lysophospholipids were predominately found in *C. lacinia* samples, which suggested that they were essential metabolites during pupal and adult stages. These findings provide insights into the natural products diversity of this plant-insect interaction and contribute to uncovering its ecological roles.

## 1. Introduction

The integration of ecological and chemical researches has enlightened how plant-insect interactions shape community dynamics and ecosystem evolution [1,2]. Recently, plant phytochemical diversity has been directly and positively correlated with the diversity of plant-associated insects [3]. In this context, the metabolomics approach is a valuable tool for unraveling biological interfaces, including the ecological bases for interactions among metabolites and the interplay between plants and insects [4,5,6,7].

Secondary metabolites (e.g., alkaloids, terpenoids and glucosinolates) have been extensively studied as important plant defensive metabolites, while the role that primary metabolites play in plant-insect interactions remains less explored [8]. Seasonal and spatial effects on plant secondary metabolite contents were associated with variation in the concentrations of chlorogenic acids and flavonoids [9]. Additionally, plants that were collected at the interface between two forest ecosystems exhibited an amplification of defensive compounds, which was related to sesquiterpene lactones (STL) content and then confirmed by the maximal cytotoxic activity [10]. Thus, studies that are based on several analytical platforms for the exploration of primary and secondary metabolites are essential for expanding the coverage of the analysis and to enable a more comprehensive understanding of these interactions [11,12]. 

*Tithonia diversifolia* (Hemsl.) A. Gray belongs to Heliantheae tribe (Asteraceae family) and it is chemically characterized by its ability to biosynthesize flavonoids, hydroxycinnamic acid derivatives, and STL, especially heliangolides, furanoheliangolides, and eudesmanolides [13,14,15]. The leaves of *T. diversifolia* present several ethnobotanical uses and biological activities (e.g., anti-inflammatory, antimalarial, analgesic, and antimicrobial activities), which are associated with their constituents [16,17]. *Chlosyne lacinia* (Geyer), popularly known as bordered path or “sunflower caterpillar”, is an oligophagous insect that feeds mostly on plants belonging to Heliantheae tribe. Butterfly wings are mainly black with yellow-orange spots of varying sizes [18,19,20]. Its larvae pass through five instars and they are usually black with dorsal orange stripes, however color polymorphism is significant within populations [21,22]. In Brazil, *T. diversifolia* leaves herbivory by *C. lacinia* mainly occurs from April to June (autumn) [23]. 

Herein, we explored the plant-insect interaction between *T. diversifolia* and *C. lacinia* by means of insect performance bioassay, untargeted LC-MS^n^, and molecular networking analyses. We thereby unraveled the natural products diversity in this plant-insect interaction and investigated the influence of the plant metabolites on the insect metabolism. 

## 2. Results and Discussion

### 2.1. C. lacinia Development

*C. lacinia* larvae that were fed with *T. diversifolia* leaves were reared in the laboratory (16/8 h of light/dark; 25 ± 2 °C) and exhibited a survival rate of 100%. Larvae passed through five instars and each took about four days (Table 1), as expected from previous reports [18,20]. Pupation occurred for approximately eight days and all the pupae were able to complete metamorphosis to the adult phase. *C. lacinia* butterflies lived for almost 12 days. During the adult life stage, butterflies mated and later laid their eggs on *T. diversifolia* leaves.

### 2.2. Natural Products Diversity

The chemical profiles of *T. diversifolia* samples (leaves and non-consumed abaxial surface) and *C. lacinia* samples (feces, larvae, pupae, butterflies, and eggs) were clearly distinct (Supplementary Material, Figures S1 and S2). In general, *T. diversifolia* samples and *C. lacinia* feces exhibited polar compounds as the major ones, especially STL and flavonoids. *C. lacinia* pupae, butterflies, and eggs were characterized by the presence of medium polarity compounds, mainly lysophospholipids. Besides, *C. lacinia* feces and larvae showed medium polarity compounds, as well as low polarity unidentified compounds.

**Figure 1:**
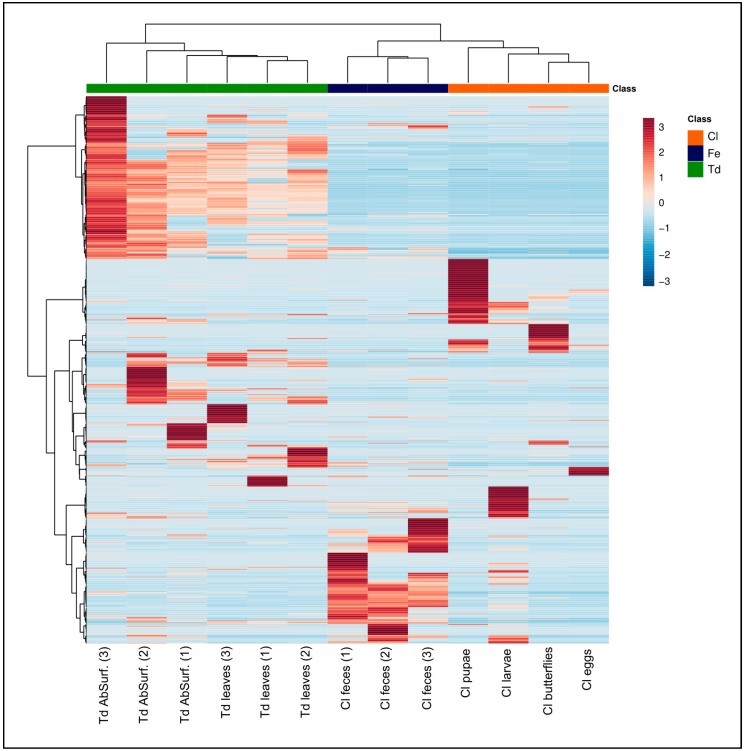
Heatmap based on mass features obtained by LC-MS^n^ (IT) in positive and negative ionization modes. Td, *T. diversifolia* (green); Cl, *C. lacinia* (orange); Fe, feces (dark blue); AbSurf., abaxial surface. Numbers in parentheses represent the replicates.

The heatmap that is based on mass features showed that the *T. diversifolia* samples were clustered apart from *C. lacinia*, which indicated that there were significant chemical differences between plant and insect (Figure 1). Among *C. lacinia* samples, feces samples exhibited several unique metabolites and they were clustered apart from other *C. lacinia* samples (larvae, pupae, butterflies, and eggs), which exhibited some exclusive metabolites but also shared some.

**Figure 2:**
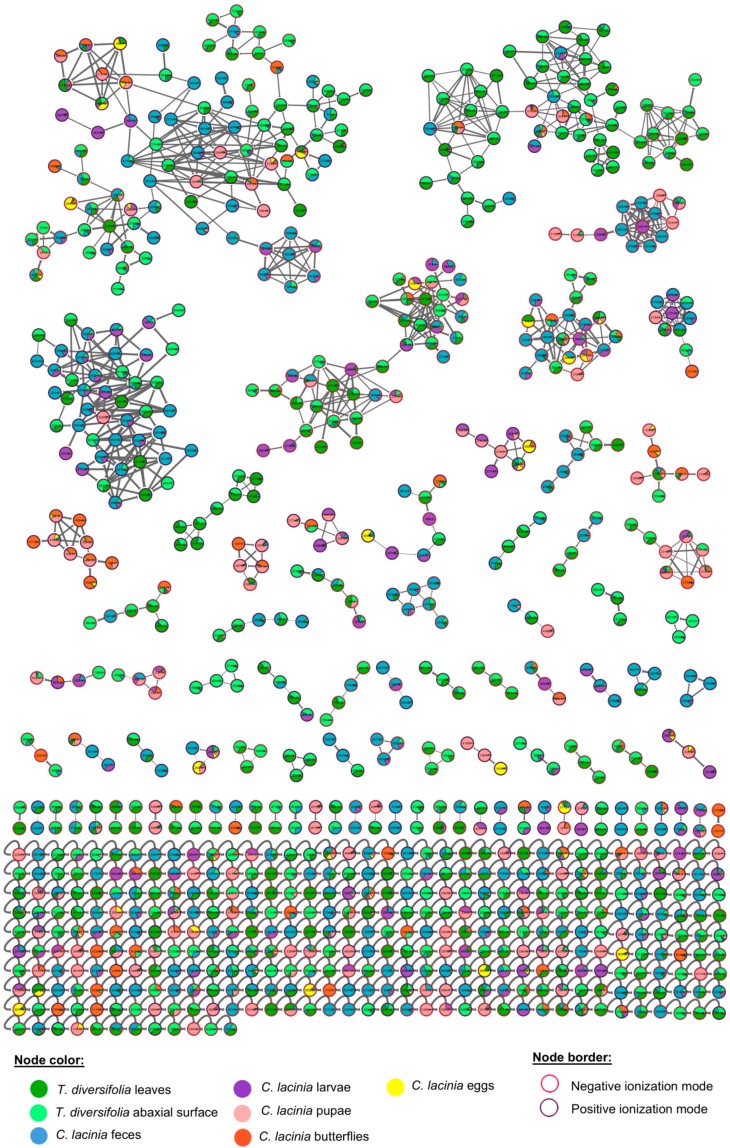
Molecular networking of *T. diversifolia* and *C. lacinia* samples analyzed by LC-MS^n^ (IT) in the positive and negative ionization modes. Nodes represent detected compounds and are colored according to the type of sample. Borders of the nodes are colored according to the ionization mode in which the mass feature was obtained. Edges between nodes represent molecular structural similarity between compounds. Nodes without GNPS spectral library matching with other nodes are represented as self-loops (bottom), i.e., edges from a vertex to itself.

Molecular networking was built with the MS data that were generated in positive and negative ionization modes, aiming to better represent the chemical diversity in this plant-insect interaction and to allow the annotation of as many compounds as possible (Figure 2) [24]. Searching against the GNPS library and previous literature reports, we were able to putatively annotate 28 compounds, according to the minimum standards that are required for level 2 non-novel metabolite identification proposed by Sumner et al. (2007) (Table 2) [25,26]. The spectrometric and spectroscopic information that were used to annotate these compounds were presented at Supplementary Material (Table S1).

**Figure 3:**
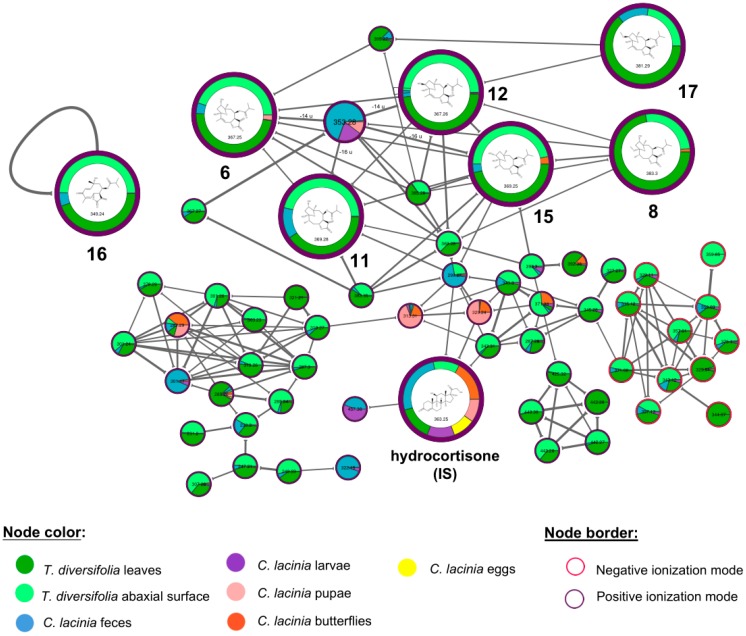
Molecular networking highlighting the cluster of annotated sesquiterpene lactones. Nodes are colored according to the type of sample. Borders of the nodes are colored according to the ionization mode in which the mass feature was obtained. IS, internal standard.

Seven furanoheliangolides-type STL, typical of Heliantheae species, were putatively annotated (**6**, **8**, **11**, **12**, **15**, **16**, and **17**). We were able to identify these STL with a high confidence level when considering the GNPS information with the main fragmentation pathways and diagnostic product ions described in the literature for furanoheliangolides [27]. STL were mainly detected in *T. diversifolia* leaves and abaxial surface (Figure 3). They were also found in *C. lacinia* samples, especially *C. lacinia* feces. Hydrocortisone, which is used as internal standard, was clustered together with the annotated STL. This proximity can be explained by the same biosynthetic origin that these compounds share (mevalonate/methylerythritol phosphate pathway) [28,29].

STL are representative secondary metabolites of Asteraceae family and exhibit particular distribution within its tribes. Several chemotaxonomic studies have applied the differential incidence of their skeleton subtype to deduce taxonomic relationships [30,31,32]. For instance, Vernonieae species are characterized by the presence of glaucolides, hirsutinolides, furanoheliangolides, and eremantholides; the Eupatorieae tribe is characterized by guaianolides, heliangolides, and mikanolides; and, the Heliantheae tribe, which comprises *T. diversifolia*, is characterized by eudesmanolides, furanoheliangolides, heliangolides, and melampolides [33,34]. More than their chemotaxonomic significance, STL play important roles in plant protection against herbivores and microorganisms, as well as in plant growth inhibition [35]. 

Field observations have shown that, during their early instars, *C. lacinia* larvae exclusively fed on the adaxial surface of *T. diversifolia* leaves [23]. This finding was associated with a behavioral strategy to avoid or reduce the intake of STL-rich glandular trichomes present in the abaxial surface of *T. diversifolia*, which may be toxic to *C. lacinia*. We found out that several STL were detected in *C. lacinia* feces during the third, fourth, and fifth larval instars, which indicated that *C. lacinia* caterpillars ingested and excreted them. Additionally, the relative abundances of some STL were higher in the whole leaves of *T. diversifolia* rather than the non-consumed abaxial surface. Thus, our results suggested that most likely *C. lacinia* larvae exclusively feeding on *T. diversifolia* adaxial surface during their early instars is not related with avoiding toxic metabolite strategy.

Previous studies have suggested that glaucolide- and hirsutinolide-type STL may be toxic to *C. lacinia* larvae, while the larvae may have developed tolerance mechanisms to deterrent compounds that are present in *T. diversifolia* (especially heliangolide- and furanoheliangolide-type STL) [36]. Herein, we only annotated STL typical of Heliantheae tribe (furanoheliangolide-type), which, as expected, were not detrimental to *C. lacinia* development. According to Ahern & Whitney (2014), the stereochemistry of the lactone ring (*cis*- or *trans*-fused) in STL was associated with herbivore resistance and plant fitness. Plants that exhibited xanthanolide-type STL with *cis*-fused lactone ring suffered higher levels of herbivore damage than plants with *trans*-fused STL [37]. Thus, the difference between the lactone moiety in glaucolide/hirsutinolide-type STLs (with endocyclic double bond) and heliangolide/furanoheliangolide-type STL (*trans*-fused *γ*-lactone ring) might explain the preference of *C. lacinia* for Heliantheae species. However, further studies, including a wider variety of STL skeletons, are required to better understand if specific subtypes are responsible for toxic and/or deterrent activity of plants and how they influence herbivore-feeding behavior. 

Additionally, by correlating the putatively annotated STL with mass differences of unknown metabolite nodes in the molecular networking, we annotated a putative tagitinin derivative STL lacking one OH group (*m*/*z* 353), which was predominantly detected in *C. lacinia* samples (feces, larvae and pupae) (Figure 3). The exact position of the missing OH could not be attributed. Thus, our results suggested that dehydroxylation may have been a potential mechanism for the excretion of STL, since a putative dehydroxylated STL was mostly detected in *C. lacinia* feces.

STL usually act as plant defensive compounds against insects through an unspecific mechanism of alkylation. The toxic effects of STL on insects were associated with morphological deformities, delayed molting, and impaired metamorphosis [38]. According to Isman (1985), some insects have developed tolerance mechanisms for toxic STL throughout the adaptation of the integument and alimentary canal, which provide an effective physical barrier for the absorption of STL and reduce their bioavailability [39]. Our data agreed with these findings, since the STL were mainly detected in *C. lacinia* feces in their intact form, which suggested that they were not absorbed from the insect digestive canal. 

**Figure 4:**
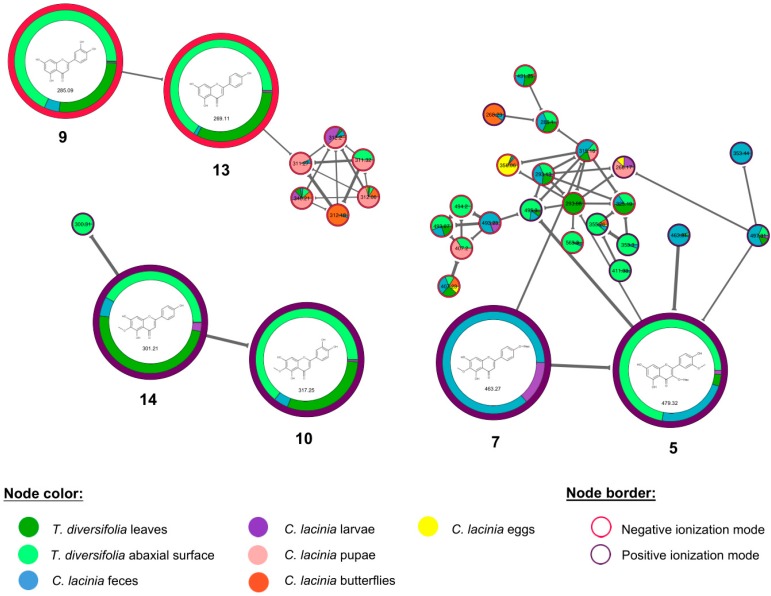
Molecular networking highlighting the cluster of annotated flavonoids. Nodes are colored according to the type of sample. Borders of the nodes are colored according to the ionization mode in which the mass feature was obtained.

Six flavonoids, including four flavones (**9**, **10**, **13**, and **14**) and two flavonoids *O*-glycosides (**5** and **7**), were also putatively annotated. Flavones were found especially in *T. diversifolia* leaves and abaxial surface, while flavonoids *O*-glycosides were mainly found in *C. lacinia* feces and larvae (Figure 4). For instance, hispidulin (**14**) was found at higher relative abundance in *T. diversifolia* leaves and abaxial surface and at lower abundance in *C. lacinia* feces and larvae, while its glycosylated flavone (**7**) was exclusively found in *C. lacinia* feces and larvae. These results showed that *C. lacinia* caterpillar excreted a minor part of the consumed flavones in their intact form and a major part was metabolized and excreted as flavone *O*-glycosides.

Flavonoids are present in plant leaves and they are known by their ability to protect plant tissue against the damaging effects of ultraviolet radiation [40,41,42]. Flavonoids also play an important role in the ecology of plant-insect interactions, especially regarding insect feeding and oviposition behavior. In spite of this, some flavonoid aglycones (e.g., quercetin) were pointed out as being responsible for detrimental effects on the development of insect larvae, because they may inhibit mitochondrial ATPase and cytochrome P450-dependent mixed function oxidases [43]. Moreover, hispidulin has exhibited insect larvicidal properties [44]. This toxicity and inhibitory activity of aglycones might explain why hispidulin was metabolized to hispidulin 4′-*O*-hexoside before its excretion.

Along with, Martucci et al. (2016) suggested that glucuronyl flavonoids from *Vernonanthura phosphorica* may be toxic to *C. lacinia* larvae [36]. We found that flavone *O*-glycosides were detected mainly in *C. lacinia* feces, which suggested that glycosylation was the main metabolism mechanism for excretion of the consumed flavonoid aglycones. Besides, these findings corroborated the potentially toxic activity of glucuronyl flavonoids that was previously described, since *C. lacinia* larvae excreted them.

Host-plant selection by herbivores is widely influenced by plant chemical diversity. While several ordinary plant compounds exist, flavonoid profiles usually vary among families, genera and species. This suggests that flavonoids can modulate host selection; however, there are still few studies in this area [45,46].

**Figure 5:**
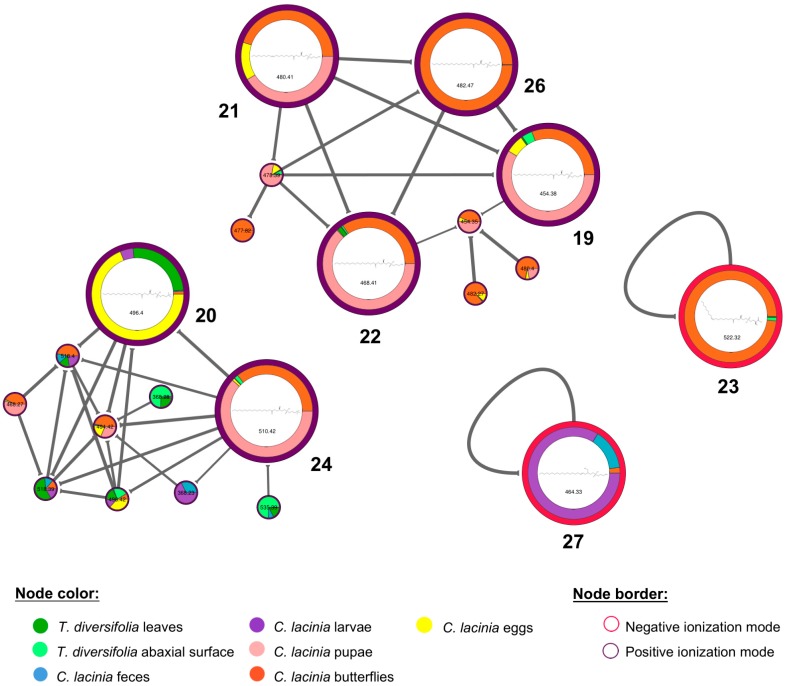
Molecular networking highlighting the clusters of annotated lysophospholipids. Nodes are colored according to the type of sample. Borders of the nodes are colored according to the ionization mode in which the mass feature was obtained.

Additionally, eight lysophospholipids were putatively annotated, including five phosphatidylethanolamines (**19**, **21**, **22**, **26**, and **27**), two phosphatidylcholines (**20** and **24**), and one phosphatidylserine (**23**). In general, these lysophospholipids were found in all samples; however, they were found in higher relative abundance in *C. lacinia* samples. Interestingly, they were clustered according to their headgroup that was attached to the phosphate group (ethanolamine, choline, or serine), forming separate clusters in the molecular networking (Figure 5).

Phospholipids are widely known by their structural function, i.e., they are the primary building blocks for cell membranes. Phosphatidylcholines and phosphatidylethanolamines are the most abundant phospholipids in insects. The proportion of each one of them is species specific and also depends on the insect physiological state [47,48]. Lysophospholipids are derivatives of phospholipids, in which one of the acyl groups have been removed by hydrolysis (containing only one fatty acid and a phosphate group). Our results suggest that lysophospholipids may play a role in *C. lacinia* metabolism, because they were predominantly found in insect samples.

Dietary lipid demands vary substantially between insect species. Usually, linoleic or linolenic acids adequately satisfy this nutritional demand. The lack of essential lipids may cause several developmental and reproduction deformations. Many insects are able to biosynthesize lipids; hence, part of the required lipids is obtained from insect diet and the other part from insect lipogenic ability [49,50]. The ability of *C. lacinia* to synthesize lipids was evidenced herein, since a greater variety of lysophospholipids was detected in *C. lacinia* samples (especially pupae and butterflies) rather than *T. diversifolia* leaves. Additionally, lysophospholipids were found at higher abundance in *C. lacinia* and at minor levels in *T. diversifolia* samples. 

Over the years, lipid metabolism in insects has been extensively studied due to the importance of lipids as substrate for metamorphosis, egg development, and flight. Besides, several insect pheromones and growth hormones exhibit lipophilic character [51,52]. Recently, lysophosphatidylcholine has been associated with antimicrobial activity in adult honeybees [53]. We found out that, in general, the putatively annotated lysophospholipids were detected in higher abundance in *C. lacinia* pupae and butterflies. Taken together, our findings suggested that lysophospholipids, along with their structural function, may play important roles by protecting the insects against microorganisms, as corroborated by previous reports [53], during pupation and by acting as an energy source for diapause and metamorphosis. Moreover, 1-(9-octadecenoyl)-glycero-3-phosphoserine (**23**) and 1-stearoyl-2-hydroxy-glycero-3-phosphoethanolamine (**26**) were predominately found in *C. lacinia* butterflies, which indicated that these metabolites may be important substrates for flight in the adult life stage, as previously reported [51,52].

The amount of lipids varies considerably during insect life and between different species. Few generalizations can be drawn regarding all the members of this animal class due to several metabolic variations that are brought about by different behavioral characteristics and ecological niches. These facts emphasize the importance of gathering specific plant-insect interactions into a broad background before drawing general conclusions. 

Additionally, we putatively annotated two amino acids (**2** and **4**), two vitamins (**1** and **3**), and three fatty acid derivatives (**18**, **25**, and **28**). Tryptophan (**2**) and phenylalanine-acetyl (**4**) were found in *T. diversifolia* and *C. lacinia* samples, especially in pupae. Pantothenic acid (**1**) was detected in all samples; however, it was found at higher abundance in *C. lacinia* pupae and butterflies. Riboflavin (**3**) was found in *T. diversifolia* leaves and mainly in *C. lacinia* butterflies. These findings suggested that the annotated amino acids and vitamins were essential during all life stages, but mostly during pupation and the adult life stage. Moreover, riboflavin may be the major compound that is responsible for the characteristic yellow-orange color of butterfly wings [54].

12,13-DiHome (**18**) was detected in all samples and it may be a precursor for other lipid derivatives. Linolenoyl-tyrosine (**25**) was exclusively found in *C. lacinia* samples (feces, larvae, and pupae), which indicated that it was produced by *C. lacinia* during their development. Moreover, it was observed in this study that oleamide (**28**), which is a fatty acid primary amide, was mostly detected in *C. lacinia* eggs.

In summary, lipid derivatives were biosynthesized during the insect life cycle and they played essential functions in *C. lacinia* development. Typical STL and flavonoids from *T. diversifolia* probably act as defensive plant metabolites, protecting *T. diversifolia* against external damage (e.g., herbivores, ultraviolet radiation). Whereas, *C. lacinia* may have, over the years, developed a strategy to reduce the bioavailability of furanoheliangolide-type STL through effective barriers in the insect digestive canal, since STL were excreted in their intact form by *C. lacinia* larvae. Altogether, our findings enlightened the main metabolism mechanisms of STL, flavonoids, and lipid derivatives in *T. diversifolia* and *C. lacinia* plant-insect interaction and provided insights into the ecological roles for interactions among metabolites. 

## 3. Material and Methods

### 3.1. Experimental Design

The egg masses of *C. lacinia* were collected in the field (21.0°10.0′9.3″ S; 47.0°50.0′48.8″ W; Medicinal Plant Garden of the School of Pharmaceutical Sciences of Ribeirão Preto, University of São Paulo, Ribeirão Preto, SP, Brazil), on the adaxial surfaces of *T. diversifolia* leaves. One egg mass was selected and maintained in a rearing box (size 30 × 25 × 20 cm, plastic floor and sides, nylon mesh top) with *T. diversifolia* fresh leaves. Seven days after the egg eclosion, 20 individuals of *C. lacinia* larvae in the second instar were randomly selected and maintained in other rearing box at laboratory conditions (16/8 h of light/dark; temperature of 25 ± 2 °C). Fresh leaves of *T. diversifolia* were collected in the same place that was reported for the egg masses and provided daily until the pupal stage. Butterflies were fed with honey:water solution (1:10, *v*/*v*). 

*C. lacinia* larval development was measured by means of survival rate from larvae to pupae and conversion from pupae to butterflies.

*C. lacinia* that were fed with *T. diversifolia* leaves were sampled at different stages of development, including third instar larvae (n = 5), pupae (n = 5), butterflies (n = 5), and eggs (one egg mass). Additionally, *C. lacinia* feces were sampled at third, fourth, and fifth larval instars. For the chemical analysis, three leaves from the same *T. diversifolia* plant were collected on three different days at the Medicinal Plant Garden of the School of Pharmaceutical Sciences of Ribeirão Preto, University of São Paulo, Ribeirão Preto, SP, Brazil. Additionally, during the larval development experiment, we sampled, on three different days, the surfaces of *T. diversifolia* leaves that were not consumed by *C. lacinia* larvae (non-consumed abaxial surfaces). All of the samples were immediately frozen and stored in a −20 °C freezer.

### 3.2. Sample Preparation

Before extraction, *T. diversifolia* (leaves and non-consumed abaxial surface) and *C. lacinia* (feces, larvae, pupae, and butterflies) samples were freeze-dried for 48 h and pulverized using mortar and pestle. Extracts were prepared using 50 mg of each sample and 1 mL of MeOH:H_2_O solution (7:3, *v*/*v*) with 10 µg·mL^−1^ hydrocortisone as internal standard, followed by vortex agitation and sonication. The extracts of *T. diversifolia* samples (whole leaves and non-consumed abaxial surface) were prepared in triplicates (n = 3). Extracts of *C. lacinia* feces were separately prepared for each feces instar sampled (third, fourth, and fifth). *C. lacinia* egg extract was prepared using a single egg mass (about 100 eggs) and the previously described procedure. After centrifugation, the supernatant was filtered through a 0.2 µm PTFE membrane into HPLC vials. 

### 3.3. LC-MS^n^ Analysis

Chemical profiles of *T. diversifolia* and *C. lacinia* samples were obtained on a Shimadzu UFLC system that was coupled to an ion trap mass spectrometer (AmaZon SL, Billerica, MA, USA). Separations were achieved while using a C18 Supelco column (Ascenti^®^ Express C_18_, 5 µm, 15 cm × 3.0 mm); water with 0.1% of formic acid (A) and acetonitrile with 0.1% of formic acid (B) as mobile phase, with a flow rate of 1.0 mL·min^−1^. The gradient was 0–2 min, 10% B; 2–40 min, 10–100% B; 40–45 min, 100% B; 45–50 min, 100–10% B; 50–55 min, 10% B. Column oven was set at 40 °C and 20 µL of each sample were injected. 

The ion trap mass spectrometer was operated in positive and negative ionization modes, separately. Instrument parameters were as follows: capillary voltage, of 3.5 kV; end plate offset, 500 V; nebulizer, 60 psi, dry gas (N_2_) flow, 11 L·min^−1^; dry temperature, 350 °C; auto MS/MS acquiring data between *m*/*z* 50 and 1300, average of three spectra; enhanced resolution for scan mode and UltraScan mode for MS/MS; spectra rate acquisition, three spectra/s; exclusion of a particular ion after three spectra and released after 30 s. The mass spectrometer was controlled by Hystar software (Bruker Daltonics Inc., Billerica, MA, USA). 

### 3.4. Data Processing and Analysis

LC-MS^n^ data of the positive and negative ionization modes, previously converted to .mzXML format while using MSconvert software (Proteowizard Software Foundation, Palo Alto, CA, USA), were separately preprocessed using MzMine ^TM^ (BMC Bioinformatics, London, UK) and employing the following parameters: mass detection using the centroid algorithm, scan MS level 1 (noise level, 1.0E3), and scan MS level 2 (noise level, 1.0E2); chromatogram builder (minimum time span in minutes, 0.1; minimum height, 2E3; *m*/*z* tolerance, 0.3 *m*/*z* or 20 ppm); chromatogram deconvolution using the baseline cut-off (minimum peak height, 2E3; peak duration, 0.1 to 1.0; baseline level, 1.0E3); isotopic peak grouper (*m*/*z* tolerance, 0.3 *m*/*z* or 20 ppm; retention time tolerance in minutes, 0.2; maximum charge, 2; representative isotope, most intense) alignment using the join aligner (*m*/*z* tolerance, 0.3 *m*/*z* or 20 ppm; weight for *m*/*z*, 50; retention time tolerance, 0.3 (abs); weight for retention time, 50 and filtering using peaks list rows filter (keep only peaks with MS2 scan). After processing, the data were exported as .csv table and as .mgf file for GNPS analysis. 

The output data from positive and negative ionization modes were combined into a single .mgf file, as well as into a single .csv spreadsheet and then uploaded to the GNPS platform. Molecular networking was built using the “advanced analysis tools for feature networking” and the following parameters: precursor ion mass tolerance, 2.0 Da; fragment ion mass tolerance, 0.5 Da; minutes pairs cos, 0.6; minimum matched fragment ions, 4; maximum shift between precursors, 500 Da; network topK, 10; maximum connected component size, 100; library search minutes matched peaks, 4; score threshold, 0.7; search analogues, don’t search; maximum analog search mass difference, 100 Da; top results to report per query, 1; minimum peak intensity, 0; filter precursor window, filter; filter library, filter library; filter peaks in 500 Da window, filter; normalization per file, no norm; aggregation method for peak abundances per group, sum. Finally, molecular networking was generated and analyzed with Cytoscape version 3.8 (Institute for Systems Biology, Seattle, WA, USA).

All of the LC-MS^n^ data were deposited on the Mass spectrometry Interactive Virtual Environment (MassIVE) at https://massive.ucsd.edu/ with the identifiers MSV000082571 (positive ionization mode) and MSV000082574 (negative ionization mode).

The online platform MetaboAnalyst (https://www.metaboanalyst.ca/faces/home.xhtml) was used to generate the heatmap. The same .csv spreadsheet used in the GNPS platform (comprising mass features in the positive and negative ionization modes) was uploaded to the statistical module, while using peak intensity table as data type and samples in rows (unpaired) as format. None data filtering was applied. Sample normalization was carried out using hydrocortisone as reference feature, followed by data auto scaling. Heatmap was built selecting the following parameters: distance measure, Euclidean; clustering Algorithm, Ward; data source, normalized data; standardization, autoscale features. 

### 3.5. Chemical Analysis and Dereplication

The MS/MS spectra of molecular networking nodes were searched against GNPS spectral library and hit compounds were appointed. The compounds that matched with structurally characterized compounds from the GNPS spectral library were compared with additional information (UV data, fragmentation pattern and previous reports in the species) and then annotated. The stereochemistry of the sugar moieties that was indicated by the GNPS library was not considered in the compound annotation (i.e., sugars were characterized as hexosides, pentosides, deoxyhexosides, etc.).

## Figures and Tables

**Table 1:** Developmental stages of *C. lacinia* reared on *T. diversifolia* leaves in the laboratory and their respective duration.

Stage	1st Instar	2nd Instar	3rd Instar	4th Instar	5th Instar	Pupation	Adult Stage	Total Development
Mean ^1^	5.25	3.70	3.25	3.45	3.55	7.65	11.35	38.20
SD ^2^	1.12	0.65	0.44	0.83	0.69	0.67	1.04	0.85

^1^ mean duration of insect instars in days; ^2^ standard deviation in days.

**Table 2:** Putatively annotated compounds in *T. diversifolia* and *C. lacinia* samples.

Rt	Usual Name (InChI)	Compound Class	Samples
1.2	**pantothenic acid (1)** (1/C9H17NO5/c1-9(2,5-11)7(14)8(15)10-4-3-6(12)13/h7,11,14H,3-5H2,1-2H3,(H,10,15)(H,12,13)/t7-/s2)	vitamin	*T. diversifolia* leaves and abaxial surface; *C. lacinia* feces, larvae, pupae, butterflies and eggs
1.4	**tryptophan (2)** (1/C11H12N2O2/c12-9(11(14)15)5-7-6-13-10-4-2-1-3-8(7)10/h1-4,6,9,13H,5,12H2,(H,14,15)/t9-/s2)	amino acid	*T. diversifolia* leaves and abaxial surface; *C. lacinia* larvae, pupae and butterflies
3.2	**riboflavin (3)** (1/C17H22N4O6/c1-7-3-9-10(4-8(7)2)21(5-11(23)14(25)12(24)6-22)15-13(18-9)16(26)20-17(27)19-15/h3-4,11-12,14-15,22-25H,5-6H2,1-2H3,(H2,19,20,26,27)/t11-,12+,14-,15?/s2)	vitamin	*T. diversifolia* leaves and abaxial surface; *C. lacinia* butterflies
4.8	**phenylalanine-acetyl (4)** (1/C11H13NO3/c1-8(13)12-10(11(14)15)7-9-5-3-2-4-6-9/h2-6,10H,7H2,1H3,(H,12,13)(H,14,15)/t10-/s2)	amino acid	*T. diversifolia* leaves; *C. lacinia* larvae, pupae and butterflies
7.2	**isorhamnetin 3-*O*-hexoside (5)** (1/C22H22O12/c1-31-12-4-8(2-3-10(12)25)20-21(17(28)15-11(26)5-9(24)6-13(15)32-20)34-22-19(30)18(29)16(27)14(7-23)33-22/h2-6,14,16,18-19,22-27,29-30H,7H2,1H3/t14-,16+,18+,19-,22+/s2)	flavonoid	*T. diversifolia* leaves and abaxial surface; *C. lacinia* feces and larvae
7.6	**orizabin (6)** (1/C19H26O7/c1-9(2)16(21)25-13-7-18(5)14(20)8-19(23,26-18)10(3)6-12-15(13)11(4)17(22)24-12/h6,9,12-15,20,23H,4,7-8H2,1-3,5H3/b10-6-/t12-,13-,14+,15+,18-,19-/s2)	STL	*T. diversifolia* leaves and abaxial surface; *C. lacinia* feces and pupae
7.7	**hispidulin 4′-*O*-hexoside (7)** (1/C22H22O11/c1-30-21-12(25)7-14-16(18(21)27)11(24)6-13(32-14)9-2-4-10(5-3-9)31-22-20(29)19(28)17(26)15(8-23)33-22/h2-7,15,17,19-20,22-23,25-29H,8H2,1H3/t15-,17-,19+,20-,22-/s2)	flavonoid	*C. lacinia* feces and larvae
8.7	**1-hydroxy-3-*O*-methyltirotundin (8)** (1/C20H30O7/c1-10(2)17(22)26-14-8-19(5)15(21)9-20(24-6,27-19)11(3)7-13-16(14)12(4)18(23)25-13/h10-11,13-16,21H,4,7-9H2,1-3,5-6H3/t11-,13+,14+,15-,16-,19+,20+/s2)	STL	*T. diversifolia* leaves and abaxial surface; *C. lacinia* butterflies
9.4	**luteolin (9)** (1S/C15H10O6/c16-8-4-11(19)15-12(20)6-13(21-14(15)5-8)7-1-2-9(17)10(18)3-7/h1-6,16-19H)	flavonoid	*T. diversifolia* leaves and abaxial surface, *C. lacinia* feces
9.6	**nepetin (10)** (1S/C16H12O7/c1-22-16-11(20)6-13-14(15(16)21)10(19)5-12(23-13)7-2-3-8(17)9(18)4-7/h2-6,17-18,20-21H,1H3)	flavonoid	*T. diversifolia* leaves and abaxial surface, *C. lacinia* feces
9.9	**tagitinin A (11)** (1/C19H28O7/c1-9(2)16(21)25-13-7-18(5)14(20)8-19(23,26-18)10(3)6-12-15(13)11(4)17(22)24-12/h9-10,12-15,20,23H,4,6-8H2,1-3,5H3/t10-,12+,13+,14-,15-,18+,19+/s2)	STL	*T. diversifolia* leaves and abaxial surface; *C. lacinia* feces
10.3	**tagitinin B (12)** (1/C19H26O7/c1-9(2)16(21)25-13-7-18(5)8-14(20)19(23,26-18)10(3)6-12-15(13)11(4)17(22)24-12/h6,9,12-15,20,23H,4,7-8H2,1-3,5H3/b10-6-/t12-,13-,14+,15+,18-,19+/s2)	STL	*T. diversifolia* leaves and abaxial surface; *C. lacinia* feces
10.9	**apigenin (13)** (1S/C15H10O5/c16-9-3-1-8(2-4-9)13-7-12(19)15-11(18)5-10(17)6-14(15)20-13/h1-7,16-18H)	flavonoid	*T. diversifolia* leaves and abaxial surface, *C. lacinia* feces
11.2	**hispidulin (14)** (1S/C16H12O6/c1-21-16-11(19)7-13-14(15(16)20)10(18)6-12(22-13)8-2-4-9(17)5-3-8/h2-7,17,19-20H,1H3)	flavonoid	*T. diversifolia* leaves and abaxial surface, *C. lacinia* feces and larvae
11.4	**2-hydroxytirotundin (15)** (1/C19H28O7/c1-9(2)16(21)25-13-7-18(5)8-14(20)19(23,26-18)10(3)6-12-15(13)11(4)17(22)24-12/h9-10,12-15,20,23H,4,6-8H2,1-3,5H3/t10-,12+,13+,14+,15-,18+,19-/s2)	STL	*T. diversifolia* leaves and abaxial surface; *C. lacinia* feces and butterflies
11.7	**tagitinin C (16)** (1/C19H24O6/c1-10(2)17(21)25-15-9-19(5,23)7-6-13(20)11(3)8-14-16(15)12(4)18(22)24-14/h6-8,10,14-16,23H,4,9H2,1-3,5H3/b7-6+,11-8-/t14-,15+,16-,19-/s2)	STL	*T. diversifolia* leaves and abaxial surface; *C. lacinia* feces
14.8	**2-*O*-methyltagitinin B (17)** (1/C20H28O7/c1-10(2)17(21)26-14-8-19(5)9-15(24-6)20(23,27-19)11(3)7-13-16(14)12(4)18(22)25-13/h7,10,13-16,23H,4,8-9H2,1-3,5-6H3/b11-7-/t13-,14-,15+,16+,19-,20+/s2)	STL	*T. diversifolia* leaves and abaxial surface; *C. lacinia* feces
18.2	**12,13-DiHOME (18)** (1/C18H34O4/c1-2-3-10-13-16(19)17(20)14-11-8-6-4-5-7-9-12-15-18(21)22/h8,11,16-17,19-20H,2-7,9-10,12-15H2,1H3,(H,21,22)/b11-8-)	fatty acid derivative	*T. diversifolia* leaves and abaxial surface; *C. lacinia* feces, larvae, pupae, butterflies and eggs
22.1	**1-hexadecanoyl-glycero-3-phosphoethanolamine (19)** (1/C21H44NO7P/c1-2-3-4-5-6-7-8-9-10-11-12-13-14-15-21(24)27-18-20(23)19-29-30(25,26)28-17-16-22/h20,23H,2-19,22H2,1H3,(H,25,26)/t20-/s2)	lysoPL	*T. diversifolia* leaves and abaxial surface; *C. lacinia* pupae, butterflies and eggs
22.5	**1-palmitoyl-glycerol-3-phosphorylcholine (20)** (1/C24H50NO7P/c1-5-6-7-8-9-10-11-12-13-14-15-16-17-18-24(27)30-21-23(26)22-32-33(28,29)31-20-19-25(2,3)4/h23,26H,5-22H2,1-4H3/p+1/t23-/s2)	lysoPL	*T. diversifolia* leaves; *C. lacinia* larvae, butterflies and eggs
22.9	**1-(9-octadecenoyl)-glycero-3-phosphoethanolamine (21)** (1/C23H46NO7P/c1-2-3-4-5-6-7-8-9-10-11-12-13-14-15-16-17-23(26)29-20-22(25)21-31-32(27,28)30-19-18-24/h9-10,22,25H,2-8,11-21,24H2,1H3,(H,27,28)/b10-9-/t22-/s2)	lysoPL	*C. lacinia* pupae, butterflies, eggs
23.6	**1-heptadecanoyl-glycero-3-phosphoethanolamine (22)** (1/C22H46NO7P/c1-2-3-4-5-6-7-8-9-10-11-12-13-14-15-16-22(25)28-19-21(24)20-30-31(26,27)29-18-17-23/h21,24H,2-20,23H2,1H3,(H,26,27)/t21-/s2)	lysoPL	*T. diversifolia* leaves; *C. lacinia* pupae and butterflies
23.8	**1-(9-octadecenoyl)-glycero-3-phosphoserine (23)** (1/C24H46NO9P/c1-2-3-4-5-6-7-8-9-10-11-12-13-14-15-16-17-23(27)32-18-21(26)19-33-35(30,31)34-20-22(25)24(28)29/h9-10,21-22,26H,2-8,11-20,25H2,1H3,(H,28,29)(H,30,31)/b10-9-/t21-,22+/s2)	lysoPL	*T. diversifolia* leaves and abaxial surface; *C. lacinia* butterflies
24.2	**1-heptadecanoyl-glycero-3-phosphocholine (24)** (1/C25H52NO7P/c1-5-6-7-8-9-10-11-12-13-14-15-16-17-18-19-25(28)31-22-24(27)23-33-34(29,30)32-21-20-26(2,3)4/h24,27H,5-23H2,1-4H3/p+1/t24-/s2)	lysoPL	*T. diversifolia* leaves and abaxial surface; *C. lacinia* pupae, butterflies and eggs
24.4	**linolenoyl-tyrosine (25)** (1/C27H39NO4/c1-2-3-4-5-6-7-8-9-10-11-12-13-14-15-16-17-26(30)28-25(27(31)32)22-23-18-20-24(29)21-19-23/h3-4,6-7,9-10,18-21,25,29H,2,5,8,11-17,22H2,1H3,(H,28,30)(H,31,32)/b4-3-,7-6-,10-9-/t25-/s2)	fatty acid derivative	*C. lacinia* feces, larvae and pupae
25.3	**1-stearoyl-2-hydroxy-glycero-3-phosphoethanolamine (26)** (1/C23H48NO7P/c1-2-3-4-5-6-7-8-9-10-11-12-13-14-15-16-17-23(26)29-20-22(25)21-31-32(27,28)30-19-18-24/h22,25H,2-21,24H2,1H3,(H,27,28)/t22-/s2)	lysoPL	*C. lacinia* butterflies
26.3	**phosphatidylethanolamine lyso alkenyl 18:0 (27)** (1/C23H48NO6P/c1-2-3-4-5-6-7-8-9-10-11-12-13-14-15-16-17-19-28-23(21-25)22-30-31(26,27)29-20-18-24/h17,19,23,25H,2-16,18,20-22,24H2,1H3,(H,26,27)/b19-17+)	lysoPL	*C. lacinia* feces, larvae and butterflies
29.3	**oleamide (28)** (1S/C18H35NO/c1-2-3-4-5-6-7-8-9-10-11-12-13-14-15-16-17-18(19)20/h9-10H,2-8,11-17H2,1H3,(H2,19,20)/b10-9-)	fatty acid derivative	*T. diversifolia* leaves and abaxial surface; *C. lacinia* butterflies and eggs

Rt, retention time in minutes; STL, sesquiterpene lactone; lysoPL, lysophospholipids. Compound structures were presented in Figure 3, Figure 4 and Figure 5.

## Supplementary Materials

The following are available online, Figure S1: Base peak chromatograms of *T. diversifolia* and *C. lacinia* samples analyzed in the positive ionization mode title, Figure S2: Base peak chromatograms of *T. diversifolia* and *C. lacinia* samples analyzed in the negative ionization mode, Table S1: UV and MS spectral data of the annotated compounds in *T. diversifolia* and *C. lacinia* samples.
