# Supplementary material for: Natural Products Diversity in Plant-Insect Interaction between Tithonia diversifolia (Asteraceae) and Chlosyne lacinia (Nymphalidae)
Source: Molecules. 2019 Aug 28;24(17):3118. doi: 10.3390/molecules24173118 (PMC6749194; doi:10.3390/molecules24173118)
Supplement: Supplementary file 1 [file molecules-24-03118-s001.zip › supplementary/Suppl. Material Natural products diversity Td e Cl (11-02-19).docx]

**Supplementary Material**

Marília Elias Gallon^1^; Eduardo Afonso Silva-Junior^1^; Juliano Geraldo Amaral^2^; Norberto Peporine Lopes^1^; Leonardo Gobbo-Neto^1^

1- Núcleo de Pesquisa em Produtos Naturais e Sintéticos, School of Pharmaceutical Sciences of Ribeirão Preto, University of São Paulo (USP), Av. do Café s/n°, 14040-903, Ribeirão Preto, SP, Brazil.

2- Instituto Multidisciplinar em Saúde, Universidade Federal da Bahia, BA, Brazil.

**
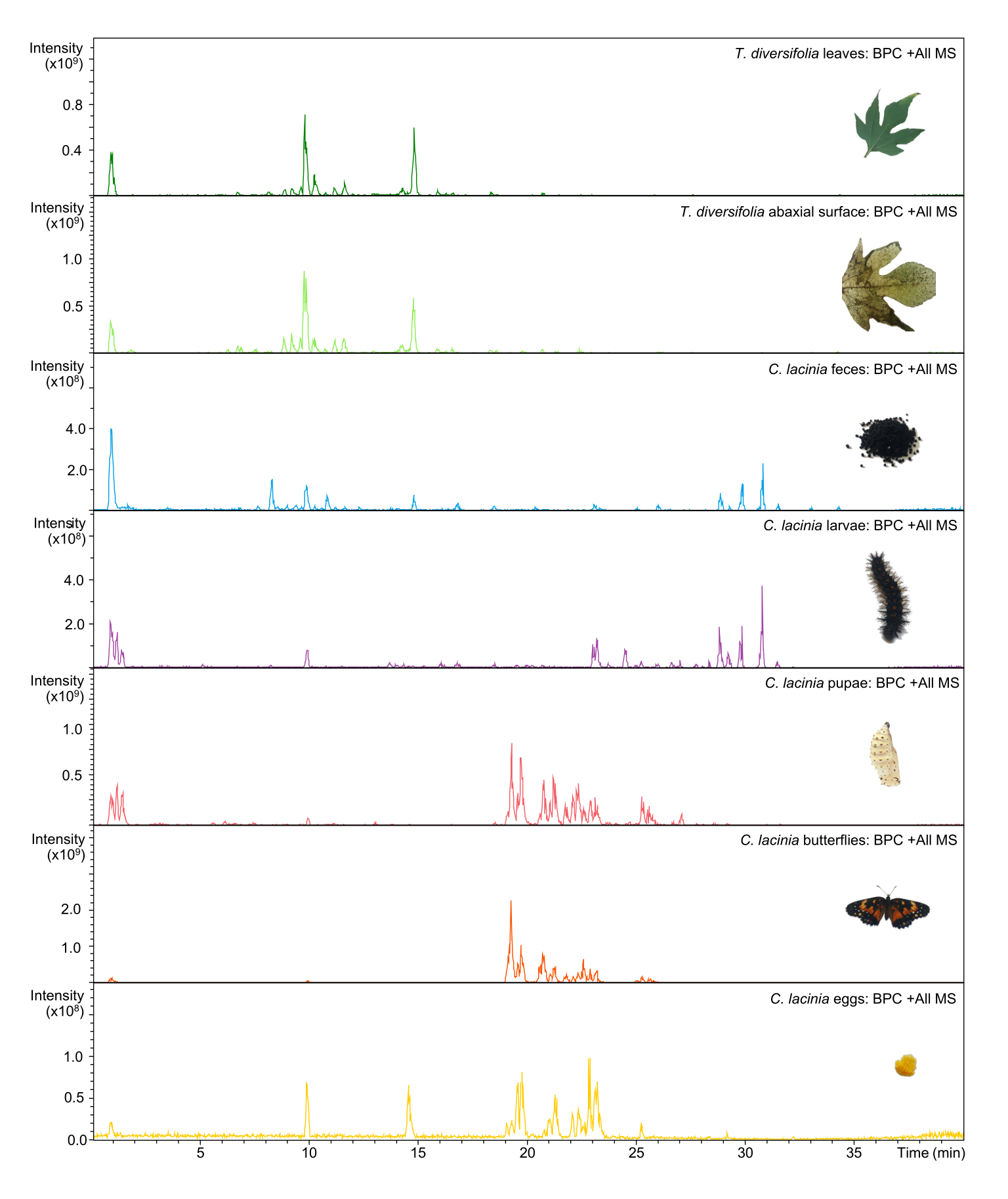
**

**Figure S1.** Base peak chromatograms of *T. diversifolia* and *C. lacinia* samples analyzed in the positive ionization mode

**
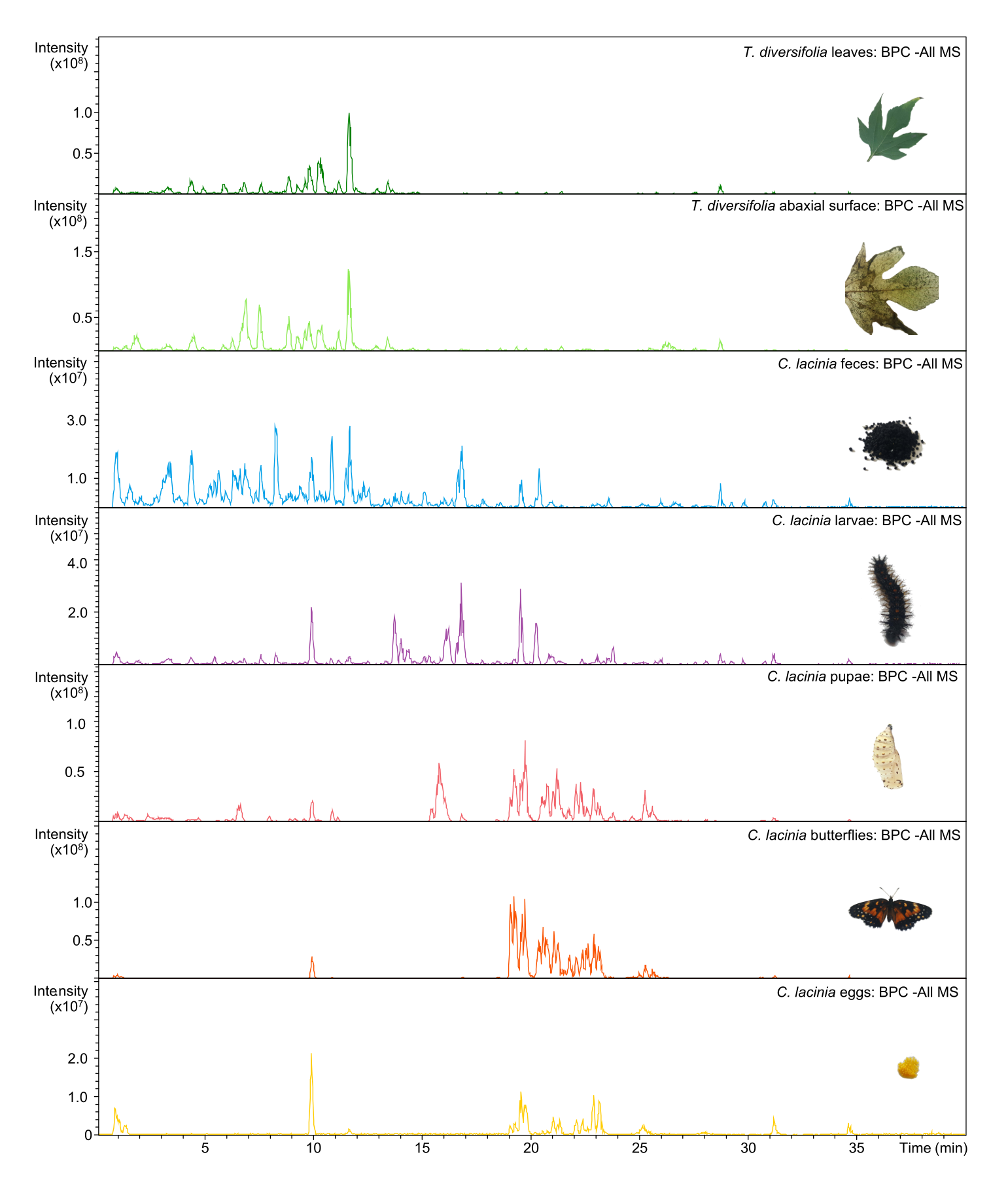
**

**Figure S2.** Base peak chromatograms of *T. diversifolia* and *C. lacinia* samples analyzed in the negative ionization mode

**Table S1:** UV and MS spectral data of the annotated compounds in *T. diversifolia* and *C. lacinia* samples.

| **ID** | **Rt** | **Usual name** | **MF**  **(Monoisot. Mass)** | | **Positive Ionization Mode** | | | | **Negative Ionization Mode** | | | | | | **UV_max_** | | | |  |  |
| --- | --- | --- | --- | --- | --- | --- | --- | --- | --- | --- | --- | --- | --- | --- | --- | --- | --- | --- | --- | --- |
|  |  |  |  |  | Precursor mass (*m/z*) | | Fragments | | Precursor mass (*m/z*) | | | Fragments | | |  | | |  |  |  |
| 1 | 1.2 | pantothenic acid * | C_9_H_17_NO_5_  (219.1107) | | [M+H]^+^ 220.2 | | 202 bp; 184; 116; 90; 72 | | [M-H]^-^ 218.1 | | 146; 116; 88 bp | | |  | | |  |  |  |  |
| 2 | 1.4 | L-tryptophan * | C_11_H_12_N_2_O_2_  (204.0899) | |  | |  | | [M-H]^-^ 203.1 | | 186; 185; 173; 159 bp; 142; 130; 116 | | |  | | |  |  |  |  |
| 3 | 3.2 | riboflavin* | C_17_H_20_N_4_O_6_  (376.1383) | | [M+H]^+^ 377.3 | | 359; 243 bp; 216; 200; 172 | |  | |  | | |  | | |  |  |  |  |
| 4 | 4.8 | L-phenylalanine-​acetyl * | C_11_H_13_NO_3_  (207.0895) | |  | |  | | [M-H]^-^ 206.1 | | 164 bp; 147 | | |  | | |  |  |  |  |
| 5 | 7.2 | isorhamnetin 3-*O*-galactoside * | C_22_H_22_O_12_  (478.1111) | | [M+H]^+^ 479.3 | | 317 bp; 302 | |  | |  | | |  | | |  |  |  |  |
| 6 | 7.6 | orizabin * | C_19_H_26_O_7_  (366.1678) | | [M+H]^+^ 367.3 | | 349; 297; 279; 261 bp; 243 | |  | |  | | | 202, 226 | | |  |  |  |  |
| 7 | 7.7 | hispidulin 4'-*O*-glucopyranoside * | C_22_H_22_O_11_  (462.1162) | | [M+H]^+^ 463.3 | | 301 bp; 286 | |  | |  | | |  | | |  |  |  |  |
| 8 | 8.7 | 1-hydroxy-3-*O*-methyltirotundin * | C_20_H_30_O_7_  (382.1991) | | [M+H]^+^ 383.3 | | 365; 295; 277 bp; 259 | |  | |  | | |  | | |  |  |  |  |
| 9 | 9.4 | luteolin* | C_15_H_10_O_6_  (286.0477) | | [M+H]^+^ 287.3 | | 269 bp; 259; 161; 153 | | [M-H]^-^ 285.1 | | 285 bp; 267; 257; 243; 241; 217; 199; 175; 151 | | |  | | |  |  |  |  |
| 10 | 9.6 | nepetin* | C_16_H_12_O_7_  (316.0583) | | [M+H]^+^ 317.2 | | 302 | | [M-H]^-^ 315.1 | | | 300 | | |  | | | | |  |
| 11 | 9.9 | tagitinin A* | C_19_H_28_O_7_  (368.1835) | | [M+H]^+^ 369.3 | | 333; 281 bp; 263; 245; 235; 217 | |  | | |  | | |  | | | | |  |
| 12 | 10.3 | tagitinin B* | C_19_H_26_O_7_  (366.1678) | | [M+H]^+^ 367.3 | | 349; 331; 279; 261 | |  | | |  | | |  | | | | |  |
| 13 | 10.9 | apigenin* | C_15_H_10_O_5_  (270.0528) | | [M+H]^+^ 271.3 | | 247; 229; 225; 211; 175 | | [M-H]^-^ 269.1 | | | 269 bp; 241; 225; 201; 183; 181; 151; 149 | | |  | | | | |  |
| 14 | 11.2 | hispidulin* | C_16_H_12_O_6_  (300.0634) | | [M+H]^+^ 301.2 | | 286 | | [M-H]^-^ 299.1 | | | 284 | | |  | | | | |  |
| 15 | 11.4 | 2-hydroxitirotudin* | C_19_H_28_O_7_  (368.1835) | | [M+H]^+^ 369.2 | | 351; 333; 281; 263; 245 bp; 227 | |  | | |  | | |  | | | | |  |
| 16 | 11.7 | tagitinin C* | C_19_H_24_O_6_  (348.1573) | | [M+H]^+^ 349.2  [M+H - H_2_O]^+^ 331.2 | | 349; 331; 261; 243; 215 | |  | | |  | | |  | | | | |  |
| 17 | 14.8 | 2-*O*-methyltagitinin B* | C_20_H_28_O_7_  (380.1835) | | [M+H]^+^ 381.2 | | 363; 311; 293; 275; 261; 243 bp | |  | | |  | | |  | | | | |  |
| 18 | 18.2 | 12,13-DiHOME* | C_18_H_34_O_4_  (314.2457) | |  | |  | | [M-H]^-^ 313.2 | | | 295 bp; 277; 195; 183 | | |  | | | | |  |
| 19 | 22.1 | 1-hexadecanoy-glycero-3-phosphoethanolamine* | | C_21_H_44_NO_7_P  (453.2855) | | [M+H]^+^ 454.4 | | 436 bp; 393; 313 | | [M-H]^-^ 452.8 | | | 316; 255 bp; 214; 196 | | |  | | | | |
| 20 | 22.5 | 1-palmitoyl-glycerol-3-phosphorylcholine* | | C_24_H_50_NO_7_P  (495.3325) | | [M+H]^+^ 496.4 | | 478; 184 bp | |  | | |  | | |  | | | | |
| 21 | 22.9 | 1-(9*Z*-octadecenoyl)-glycero-3-phosphoethanolamine* | | C_23_H_46_NO_7_P  (479.3012) | | [M+H]^+^ 480.4 | | 462 bp; 419; 339; 265 | | [M-H]^-^ 478.4 | | | 282; 281 bp; 214 | | |  | | | | |
| 22 | 23.6 | 1-heptadecanoyl-glycero-3-phosphoethanolamine* | | C_22_H_46_NO_7_P  (467.3012) | | [M+H]^+^ 468.4 | | 450 bp; 407; 327; 296 | |  | | |  | | |  | | | | |
| 23 | 23.8 | 1-(9*Z*-octadecenoyl)-glycero-3-phosphoserine | | C_24_H_46_NO_9_P  (523.2910) | |  | |  | | [M-H]^-^ 522.3 | | | 435 bp; 417; 170; 153 | | |  | | | | |
| 24 | 24.2 | 1-heptadecanoyl-glycero-3-phosphocholine* | | C_25_H_52_NO_7_P  (509.3481) | | [M+H]^+^ 510.4 | | 492 bp; 313; 184 | |  | | |  | | |  | | | | |
| 25 | 24.4 | linolenoyl-tyrosine* | | C_27_H_39_NO_4_  (441.2879) | | [M+H]^+^ 442.4 | | 396; 261; 243; 182 bp; 165; 147; 136 | |  | | |  | | |  | | | | |
| 26 | 25.3 | 1-stearoyl-2-hydroxy-glycero-3-phosphoethanolamine* | | C_23_H_48_NO_7_P  (481.3168) | | [M+H]^+^ 482.4 | | 482; 464 bp; 421; 341 | |  | | |  | | |  | | | | |
| 27 | 26.3 | phosphatidylethanolamine lyso alkenyl 18:0* | | C_23_H_48_NO_6_P  (465.3219) | |  | |  | | [M-H]^-^ 464.3 | | | 404; 403 bp; 268; 267; 197; 196; 153; 140 | | |  | | | | |
| 28 | 29.3 | oleamide* | | C_18_H_35_NO  (281.2718) | | [M+H]^+^ 282.3 | | 265; 247 bp; 223; 219; 198; 191; 184; 177; 167; 163; 156; 149; 142; 135; 121; 111; 109; 97; 95 | |  | | |  | | |  | | | | |

ID, peak identification; Rt, retention time in minutes; MF, molecular formula; Monoisot. Mass, monoisotopic mass; UVmax, wavelength of maximum absorption in the ultraviolet spectral region; sh, shoulder; bp, base peak; * HRMS used for confirmation of compounds.
