# Supplementary figures and images for: Natural Products Diversity in Plant-Insect Interaction between Tithonia diversifolia (Asteraceae) and Chlosyne lacinia (Nymphalidae)
Source: Molecules. 2019 Aug 28;24(17):3118. doi: 10.3390/molecules24173118 (PMC6749194; doi:10.3390/molecules24173118)

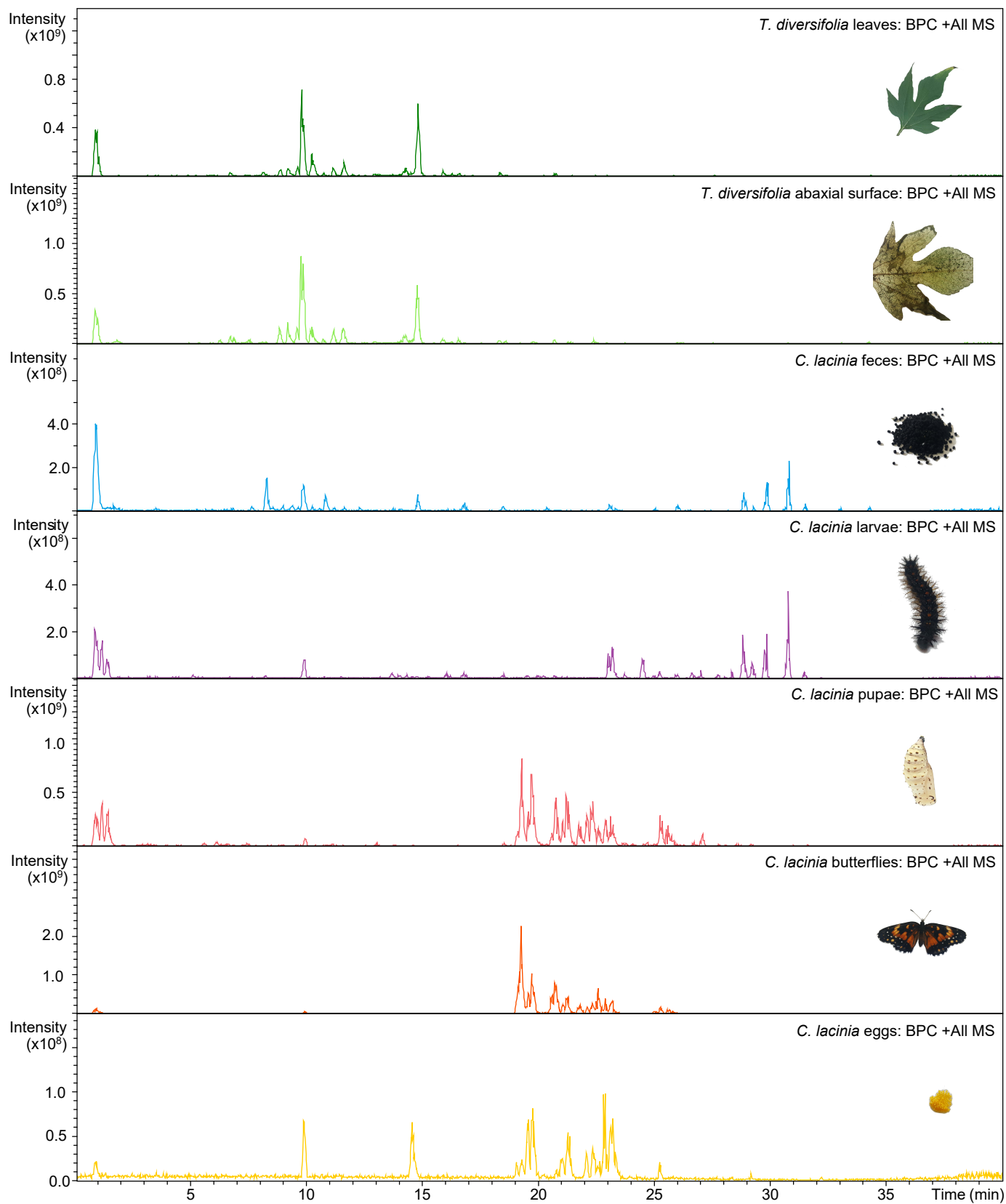

Supplement: Supplementary file 1 [file molecules-24-03118-s001.zip › supplementary/Figure S1.pdf]

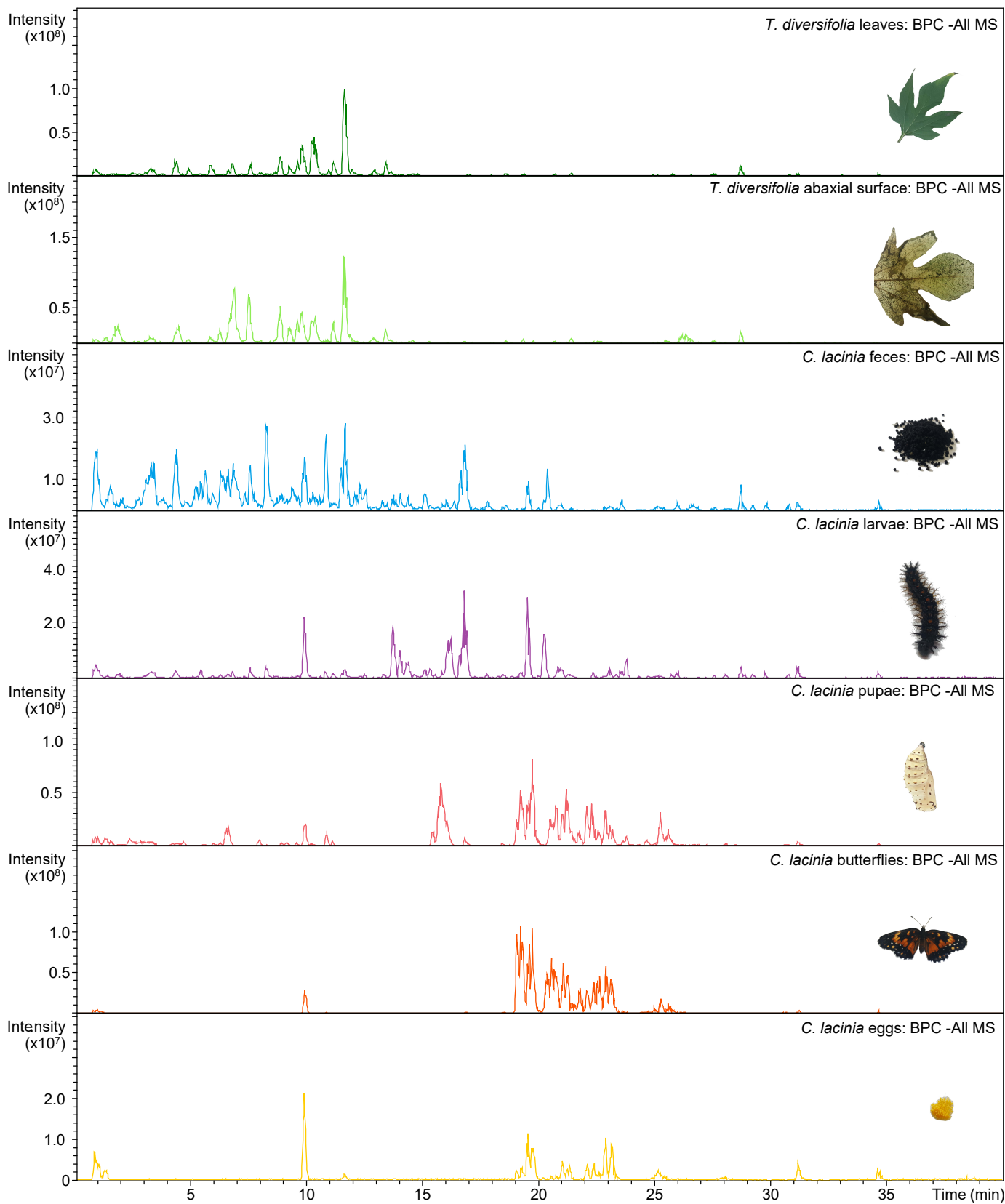

Supplement: Supplementary file 1 [file molecules-24-03118-s001.zip › supplementary/Figure S2.pdf]
